# Fusing Local Shallow Features and Global Deep Features to Identify Beaks

**DOI:** 10.3390/ani13182891

**Published:** 2023-09-12

**Authors:** Qi He, Qianqian Zhao, Danfeng Zhao, Bilin Liu, Moxian Chu

**Affiliations:** 1 College of Information Technology, Shanghai Ocean University, Shanghai 201306, China; qihe@shou.edu.cn (Q.H.); m210911504@st.shou.edu.cn (Q.Z.); dfzhao@shou.edu.cn (D.Z.); 2 College of Marine Sciences, Shanghai Ocean University, Shanghai 201306, China; m220250829@st.shou.edu.cn; 3 The Key Laboratory of Sustainable Exploitation of Oceanic Fisheries Resources, Ministry of Education, Shanghai Ocean University, Shanghai 201306, China; 4 National Distant-Water Fisheries Engineering Research Center, Shanghai Ocean University, Shanghai 201306, China; 5 Key Laboratory of Oceanic Fisheries Exploration, Ministry of Agriculture and Rural Affairs, Shanghai 201306, China

**Keywords:** cephalopods, beaks, CNN, HOG, LBP, SVM, feature fusion

## Abstract

**Simple Summary:**

Cephalopods are not only important economic products in fisheries, but also located in the middle pyramid of the marine ecosystem, playing a role of carrying the top and bottom. Cephalopods are the meals of large marine mammals, and their soft tissues are mostly digested in the stomach, and the beaks can be retained as hard tissues of cephalopods, which are structurally stable and resistant to corrosion. Therefore, the biodiversity of cephalopods can be analyzed by studying the beaks. However, there are many difficulties in the identification of beaks, such as the high level of similarity between different species of beaks and the variability arising from the growth process. The local shallow features, namely texture features and morphological features, and the global deep features were used, and the two types of features were fused for identification. This study verifies the complementarity between the two types of features and further contributes to the progress of beak recognition, providing a new approach to analyzing the biodiversity of cephalopods.

**Abstract:**

Cephalopods are an essential component of marine ecosystems, which are of great significance for the development of marine resources, ecological balance, and human food supply. At the same time, the preservation of cephalopod resources and the promotion of sustainable utilization also require attention. Many studies on the classification of cephalopods focus on the analysis of their beaks. In this study, we propose a feature fusion-based method for the identification of beaks, which uses the convolutional neural network (CNN) model as its basic architecture and a multi-class support vector machine (SVM) for classification. First, two local shallow features are extracted, namely the histogram of the orientation gradient (HOG) and the local binary pattern (LBP), and classified using SVM. Second, multiple CNN models were used for end-to-end learning to identify the beaks, and model performance was compared. Finally, the global deep features of beaks were extracted from the Resnet50 model, fused with the two local shallow features, and classified using SVM. The experimental results demonstrate that the feature fusion model can effectively fuse multiple features to recognize beaks and improve classification accuracy. Among them, the HOG+Resnet50 method has the highest accuracy in recognizing the upper and lower beaks, with 91.88% and 93.63%, respectively. Therefore, this new approach facilitated identification studies of cephalopod beaks.

## 1. Introduction

The important role cephalopods (Mollusca: Cephalopoda) play in many marine ecosystems has been widely acknowledged [1]. Cephalopods are predators for numerous prey and are preyed upon by predators [2,3,4,5]. In particular, cephalopods are one of the main food sources for large marine predators such as whales [6], dolphins [7], and sharks [8]. Consequently, cephalopods are located in the middle of the marine trophic level pyramid, playing a significant role in the marine food chain and nutrition structure [9]. Furthermore, cephalopods are significant marine animals in economic terms, due to their short life cycles (typically 1 year), rapid growth, and abundant resources [10]. In recent decades, the development of the global cephalopod fishery industry and the production of edible cephalopods have accelerated. Research on cephalopods is beneficial for the sustainable utilization of this resource and will also increase the number of cephalopod species available for future commercial development.

The majority of our understanding of cephalopods comes from analyzing the stomach contents of their predators. The identification of cephalopods in stomach contents is typically dependent on beaks since the majority of soft tissue has been digested, but the beaks can resist digestion for as long as several months [11,12]. As the main feeding organ of cephalopods, beaks are located in the buccal mass and are divided into the upper beak and the lower beak [13,14,15,16]. The beak is one of the hard tissues in cephalopods, which has a stable structure and is resistant to corrosion [14]. In recent years, the beak has been extensively utilized for the identification of cephalopod populations [13,17] and the classification of species [18]. Therefore, a lot of research work has been devoted to improving the feature extraction and recognition methods for beaks.

In the field of computer vision, shallow features refer to extracting basic image attributes or features from image data. The common shallow features in computer vision include edge features, texture features, morphological features, color features, and so on. The morphological features of the beak are a useful tool for searching for inter- and intra-species differences in cephalopods, as well as for species identification [10]. Hence, the majority of research on the classification of beaks has centered on refining methods for extracting morphological features. The research on deriving the morphological features of the beak focuses primarily on the calibration of feature points and the extraction of feature parameters [19]. With the development of artificial intelligence, edge detection has been applied as a basic method for image processing using computer vision in the study of beak recognition. He Q H et al. [20] extracted the contours of the beak by using the canny algorithm to assist in the calibration of feature points and extraction of feature parameters, which resulted in addressing issues such as time-consuming and labor-intensive manual measurements. Wang B Y et al. [21] proposed an improved edge detection method to extract the morphological outer contour of the beak, which can effectively distinguish signal noise and improve the accuracy of target selection, while ensuring the integrity of the contour within the error tolerance. The feature algorithm used in the above study to extract a single shallow feature of the beak is effective in beak image classification, which has the advantages of high interpretability, good performance with a small number of samples, and low computational resource requirements. This traditional method typically requires the manual design of region of interest features and feature extraction operators in the image, which fails to fully define the subtle differences in the beak and is, therefore, sensitive to changes in scale and morphology.

CNN is the most prominent deep learning method in which the multiple layers are trained and tested robustly. In recent years, deep learning has been broadly applied in various domains [22], since it autonomously extracts image features for image recognition [23,24]. Deep features are high-level feature representations that are learned from original image data by deeply learned models. These features can help computers better understand and utilize complex real-world data. Tan H Y et al. [25] extracted shallow and deep features from beaks and classified them using eight machine learning classification methods. They concluded that deep features were preferable to shallow ones for beak classification. This model has several limitations, including imbalance and a small sample size, as well as a single beak view and a limited number of morphological features in morphological shape descriptors (MSDs). Deep learning methods based on the convolutional neural network (CNN) model have led to significant breakthroughs in various fields, as they can achieve the extraction of complex target features to some extent and also reduce the errors arising from human-defined features. However, the CNN model requires a large amount of labeled data and a long training time to fully learn and represent complex features within the image data. Also, its performance may be limited in the face of an insufficient amount of beak data. Therefore, shallow feature algorithms and deep feature methods have advantages and disadvantages and differ in their representation of features.

Based on the preceding analysis, we have reason to believe that traditional algorithms are beneficial for extracting shallow features for classifying beaks. However, the feature information extracted by a single feature descriptor is relatively limited, and the required features may not be extracted sufficiently. At the same time, the deep features contain semantic information, but due to the limited number of beak samples, the descriptor may not be able to extract all the necessary details. Therefore, we tried to improve accuracy by describing multiple features of the image and achieving a complementary means of feature information. This study proposes, for the first time, a recognition method based on fusing global deep features with local shallow features in the field of beak research. The study included four cephalopod species, namely *Dosidicus gigas* (*D. gigas*), *Illex argentinus* (*I. argentinus*), *Eucleoteuthis luminosa* (*E. luminosa*), and *Ommastrephes bartramii* (*O. bartramii*), which provided images of upper and lower beaks. Initially, the histogram of the orientation gradient (HOG) and the local binary pattern (LBP) feature descriptors were employed to derive the morphological and texture features from the beak image. Meanwhile, we selected the optimal CNN model for deep feature extraction, including the VGG16 [26], InceptionV3 [27], and Resnet series [28]. Next, two types of local shallow features and global deep features were fused separately to highlight the details of the features, and the support vector machine (SVM) classifier was utilized for classification. This method will facilitate the development of beak recognition and provide a new and feasible strategy for future cephalopod biodiversity studies.

## 2. Materials and Methods

### 2.1. Materials

In this study, we collected samples of four oceanic cephalopods targeted for fisheries, including *D. gigas*, *I. argentinus*, *E. luminosa*, and *O. bartramii* (Table 1). Among them, *D. gigas*, *I. argentinus*, and *O. bartramii* were obtained by handfishing from squid boats, and *E. luminosa* was caught using trawl nets. Species identification was confirmed with reference to Jereb P et al. [29]. These specimens were selected to represent the diversity of unique morphological groups and size classes during the sampling process. The collected samples were frozen immediately upon arrival at the laboratory and the beaks were peeled off and stored in bottles containing 75% ethanol. A total of 200 beak samples were obtained.

### 2.2. Image Acquisition

**Figure 1:**
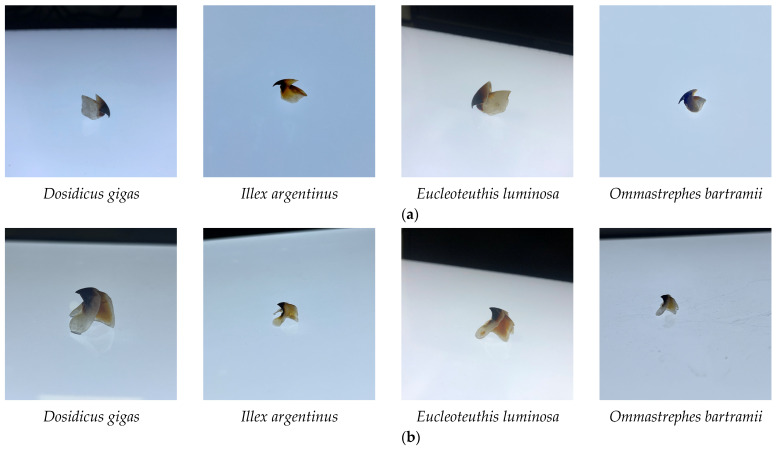
Four species of beaks. (**a**) Represents the upper beak image. (**b**) Represents the lower beak image.

We collected digital images of the beak. First, the beak sample was placed in the center of the white light board, and a smartphone was used as the shooting instrument to capture images from multiple angles, including the top view, left view, right view, front view, etc. (Figure 1). The project gathered 4000 images to satisfy the training requirements of the CNN model. The original image resolution was 3020 px × 3020 px and all images were saved in JPEG format. Then, images were input into the model for feature extraction and resized according to the image input standards.

### 2.3. Partition Dataset

In shallow feature extraction models, the dataset of upper and lower beaks for each species was split into training and testing sets according to an 80% and 20% ratio. A total of 20% of the dataset was used as the testing set. The remaining 80% of the beak dataset was randomly split into 80% for training and 20% for the validation set in the CNN model. After each training iteration, the validation set serves as a preliminary evaluation of the learning architecture. Once the CNN model had been trained, the parameters (network weights) were stored and used to evaluate the performance of the testing set. There was zero overlap between the training set, validation set, and testing set.

### 2.4. Data Augmentation

**Figure 2:**
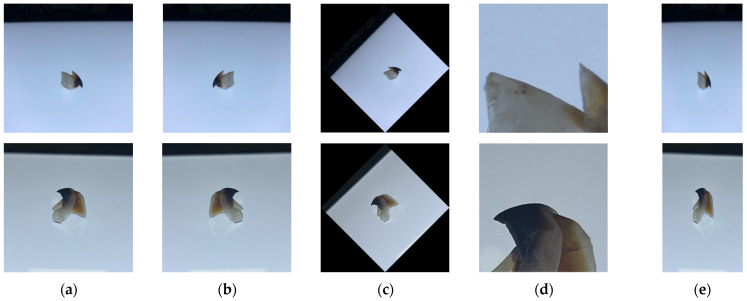
Data enhancement example: (**a**) original image; (**b**) flip the image horizontally; (**c**) rotate the image 45 degrees to the left; (**d**) randomly crop the image; (**e**) randomly change the width and length of the image.

It is a generally accepted notion that a bigger dataset results in better deep learning models [30,31]. Data augmentation is a frequently employed technique in deep learning that generates new training samples by expanding and transforming the original data. An affine transformation was used for data augmentation (Figure 2). This can be written as follows:

(1) y=ωx+b

where y represents the transformed data, ω represents the weight matrix that contains the parameters of the transformation, x is the input data, and *b* is a constant term.

The following enhanced parameters were applied:

Image flipping and rotation: by randomly determining whether or not to perform the flip and rotate operation and by randomly generating the corresponding parameters (flip direction and rotation angle).

Random crop: by selecting at random the position of a crop box on the original image and cropping it. 

Scale transformation: by generating a new aspect ratio at random and calculating the new width and height of the beak image.

### 2.5. Methods

**Figure 3:**
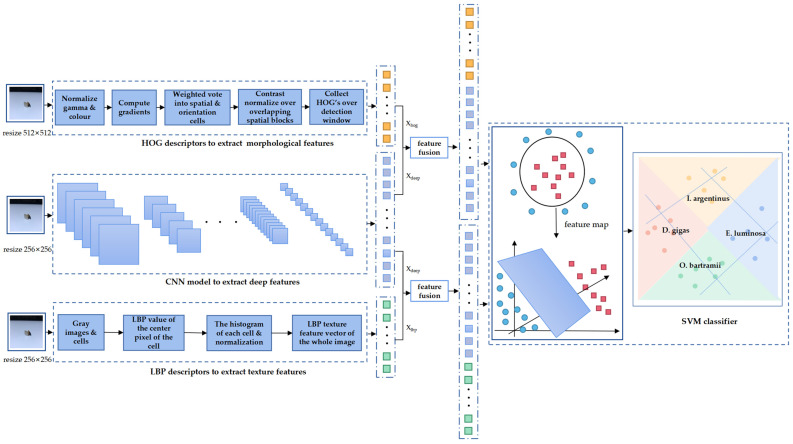
Flowchart for the classification of beaks with names *D. gigas*, *I. argentinus*, *E. luminosa*, and *O. bartramii*. x_hog_ are the HOG features, x_deep_ are the deep features and x_lbp_ is the LBP feature.

The methods for identifying the four species of beaks can be split into four main stages: (a) obtaining digital images of beaks and adjusting the image size; (b) extracting the shallow features of HOG and LBP, and using the SVM classifier to automatically classify the beak; (c) obtaining deep features through six different CNN models and classifying them; (d) selecting the deep features with the best deep model in fusion with shallow features and using the SVM classifier to identify the beaks (Figure 3). The details of the specific process steps are as follows. 

#### 2.5.1. Local Shallow Feature Extraction

##### Local Binary Patterns (LBP)

**Figure 4:**
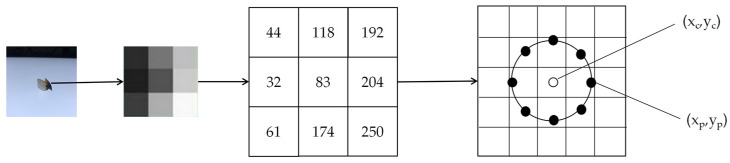
There are 8 sampling points in a 2 cm radius circular neighborhood.

LBP [32] is a descriptor used to characterize the local texture features of the image, with robust extraction capabilities for texture information. The method must be applied to image regions containing multiple points, as opposed to a single pixel. The improved LBP descriptor is employed to adapt to texture features of varying dimensions and satisfy the requirements of grayscale and rotation invariance, which replaces square neighborhoods with circular neighborhoods. In the image of the beak, the improved LBP descriptor permits random P sampling points within a circular neighborhood of radius R (Figure 4).

Expressed in terms of the formula:

(2) $$LBP(x_{c},y_{c})=\sum_{p=0}^{P-1}2^{p}s(i_{p}-i_{c})$$

where ${(x}_{c},y_{c})$ is the center pixel, *P* is the number of samples, p belongs to a number from 0 to *P*, $i_{c}$ is the gray value, $i_{p}$ is the gray value of the neighboring pixel, and S is a sign function;

(3) $$S(x)=\left\{\begin{array}{c} 1\;\;\;\;\;if\;x\geq \;0\\ 0\;\;\;\;\;\;\;\;\;\;\;\;\;else\; \end{array}\right.$$

For a given center point $\left(x_{c},y_{c}\right)$, the position of the sampling point $\left(x_{p},y_{p}\right)$ is determined using Equations (4) and (5), p∈P, *P* is the number of samples, p belongs to the number from 0 to *P*.

(4) $$x_{p}=x_{c}+R\cos\left(\frac{2p}{P}\right)$$

(5) $$y_{p}=y_{c}-R\sin\left(\frac{2\pi p}{P}\right)$$

The LBP statistical histogram is the feature vector of the beak image (Figure 3). The following is a summary of the LBP features extracted from the beak image:

1. The image of the beak is converted to a grayscale image and divided into *n* × *n* cells;
2. The central pixel of each cell is compared with *P* pixels in the circular neighborhood and the LBP value of each cell is calculated;
3. Normalize the histogram of every cell;
4. The histograms of all cells are concatenated as texture feature vectors of the whole image.

##### Histogram of Oriented Gradient (HOG)

**Figure 5:**
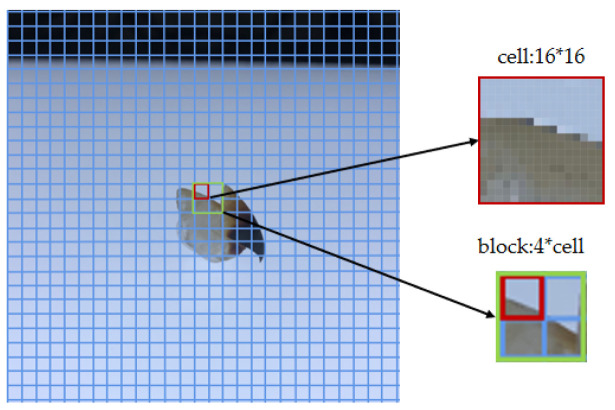
Each cell contains 16 × 16 pixel points and 4 cells form a block of the HOG descriptor.

HOG is one of the best features to capture edge or local morphological information [33] and is widely used in machine learning, pattern recognition, and image processing that uses gradient information to reflect the edge features of beak images and describe the appearance and morphology of images based on the value of local gradients (Figure 3). The following is a summary of the extraction process:

1. The beak image is grayscaled and normalized, which diminishes the effect of shadows and illumination on the image and reduces noise.
2. The gradient (including size and direction) of each pixel is calculated, and the image is divided into multiple units. The gradient calculation formula is defined as:

(6) $$G_{x}\left(x,y\right)=I\left(x+1,y\right)-I\left(x-1,y\right)$$

(7) $$G_{y}\left(x,y\right)=I\left(x,y+1\right)-I\left(x,y-1\right)$$

(8) $$\nabla G\left(X,Y\right)=\sqrt{G_{x}{\left(x,y\right)}^{2}+G_{y}{\left(x,y\right)}^{2}}$$

(9) $$theta\left(x,y\right)=arctan\left(\frac{G_{y}\left(x,y\right)}{G_{x}\left(x,y\right)}\right)$$

where $G_{x}\left(x,y\right)$ is the gradient in the *x* direction, $G_{y}\left(x,y\right)$ is the gradient in the *y* direction, $\nabla G\left(X,Y\right)$ is the gradient amplitude, $theta\left(x,y\right)$ is angle;

3. The image is separated into numerous cells. Each cell consists of C × C pixels, with N cells forming a block (Figure 5).
4. The statistical direction gradient histogram of the cell builds a block, and the feature vectors of the beak image are obtained by connecting feature vectors of all blocks.

#### 2.5.2. Deep Feature Extraction

**Figure 6:**
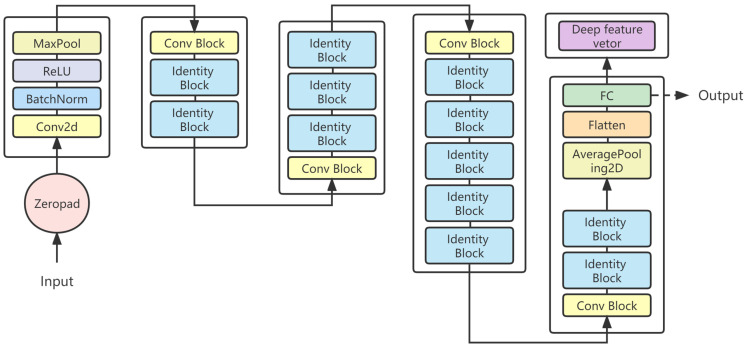
The network architecture of Resnet50 for deep feature extraction.

Deep learning is a field of machine learning which learns high-level abstractions in data by using hierarchical architectures [34]. Three typical CNN models are used to extract deep features, namely VGG16, InceptionV3, and ResNet series. ResNet can be divided into ResNet18, ResNet34, ResNet50 (Figure 6), and ResNet101 based on the network structure of different layers. CNN typically includes three fundamental operations: convolution layers, pooling layers, and full connection layers. The convolutional layer refers to the application of convolutional kernels to obtain image pixel data. The main function of the pooling layer is to reduce redundant feature data. The full connection layer acts as a classifier.

#### 2.5.3. Feature Fusion

The results of the extraction of image features frequently influence the accuracy of classification. A single type of image feature overlooks the complementarity of multiple features. Shallow features and deep features each represent different species of image features. In this study, shallow features and deep features are fused, which means deep features are fused with HOG or LBP features to form fused features, and this method effectively utilizes the relationship between various features to obtain more discriminative detailed features. The fused features are introduced through the SVM classifier to obtain the final classification result (Figure 3).

In this paper, the vector stacking fusion method is used. Assuming that $x_{hog}$ is a morphological feature, $x_{deep}$ is a deep feature and $x_{lbp}$ is a texture feature. The feature fusion expression is as follows:

(10) $$y_{1}=[x_{hog},x_{deep}]$$

(11) $$y_{2}=[x_{lbp},x_{deep}]$$

where, $y_{1}$ represents the fusion of morphological features and deep features, $y_{2}$ represents the fusion of texture features and deep features.

#### 2.5.4. Support Vector Machine (SVM)

SVM originated for binary classification problems and is a binary classification model. SVM is extensively used in species classification and is considered a representative of machine learning [35], which can solve both linearly separable and linearly nonseparable problems [36]. The classification of four species of beaks is a nonlinear problem with multi-class classifications. When addressing problems involving multiple classes, it is necessary to develop appropriate multi-class classifiers [37,38]. Therefore, the basic theory of multi-class SVM is to transform the space by introducing the kernel function K and find the optimal hyperplane in the high-dimensional feature space to maximize the distance between multi-class samples, thus transforming the nonlinear classification problem into the high-dimensional linear classification problem [36,39].

#### 2.5.5. Performance Evaluation

The confusion matrix is a performance evaluation tool presented in matrix form to measure the classification results of a model for different species. In this study, the classification was based on the true beak species and the predicted beak species, and the classification results could be classified into four different cases: true positive *(TP*), false positive (*FP*), true negative (*TN*), and false negative (*FN*). Based on the confusion matrix, a series of classification performance metrics were calculated, including accuracy, precision, recall, and *F*1-*score*. Accuracy is the ratio of the number of correctly classified samples to the total number of samples. Precision is defined as the proportion of true positives to all positives predicted by the model. Recall is the proportion of correctly predicted positive classes relative to the total number of actual positive classes. *F*1-*score* is an all-encompassing measure of precision and recall rate. The following are the definitions of these classification indicators:

(12) $$Accuracy=\frac{TP+TN}{TP+TN+FP+FN}$$

(13) $$Precision=\frac{TP}{TP+FP}$$

(14) $$Recall=\frac{TP}{TP+FN}\;$$

(15) $$F1\text{-}score=2\frac{Precision\times Recall}{Precision+Recall}$$

where, *TP* = true positive, *TN* = true negative, *FP* = false positive, *FN* = false negative.

#### 2.5.6. Experimental Parameter Settings

Based on prior knowledge of hyperparameter settings for the SVM classifier, this study chose the hyperparameter that achieves the best results for SVM (Table 2). The parameters of the multi-class SVM classifier are kernel function (K), C, and decision function morphological, correspondingly. K is set to “rbf” so that the feature data of the beak is separable in the feature space. C represents the penalty coefficient, which is the tolerance for errors. 

In addition, the relevant parameters of all deep feature extraction models include learning rate, epoch, and batch size (Table 2). The learning rate is closely related to the convergence process of the model. In this experiment, the learning rates of 0–50 epoch and 51–100 epoch are set to 1 × 10^−3^ and 1 × 10^−4^, respectively. The appropriate batch size is selected based on factors such as hardware resources, model complexity, and dataset size, so batch size = 16 in this experiment. In the actual training, the appropriate number of epochs was chosen according to the convergence of the model and the limitation of computational resources, which can determine the required eopch = 100 for complete training.

The experimental environment included computer processor Intel(R) Xeon(R) Gold 6130 CPU @ 2.10 GHz, Intel Corporation, Santa Clara, CA, USA; mainboard model YZMB-00882-104, Samsung, Seoul, SouthKorea; primary hard drive ADC55CE5-E726-44DF-A85C-CE534483DE11, Seagate Technology PLC, Dublin, Ireland; graphics card NVIDIA TITAN RTX (24,576 MB), NVIDIA Corporation, Santa Clara, CA, USA; and Python 3.8.1.

## 3. Results

### 3.1. Using Shallow Features and SVM for the Classification of Beaks

This experiment tests the classification performance of HOG and LBP on the beak test set to determine the optimal LBP and HOG features (Table 3).

When R = 1 and P = 8, the greatest classification results were obtained for the upper and lower beaks, with 53.63% and 41.88%, respectively (Table 4). 

In the HOG experiment, morphological features were extracted from beak images by adjusting the C value. The experimental results showed that when C = 64, the classification accuracy for the upper and lower beaks was 70.25% and 58.00%, respectively. When C = 32, the classification accuracy was 69.38% and 60.50%, respectively (Table 5). The two sets of experiments showed that the local morphological features extracted by the HOG descriptor more accurately express the differences between the four species of beaks.

### 3.2. Six CNN Models for Extracting Deep Features

**Figure 7:**
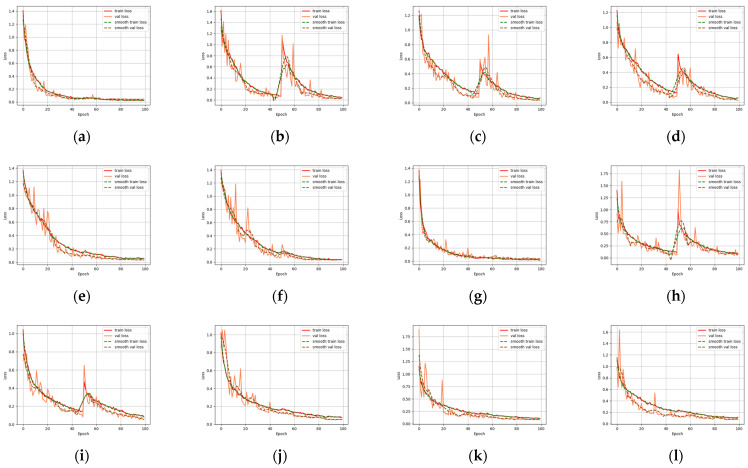
The loss function curves of the validation set and testing set derived from six CNN models, namely VGG16, InceptionV3, Resnet18, Resnet34, Resnet50, Resnet101: (**a**–**f**) represents the loss function curves for the upper beak; (**g**–**l**) represents the loss function curve for the lower beak.

The loss function curves of the training and validation sets (Figure 7) are important tools for model selection and tuning, which can help determine how well the model fits, its generalization ability, and the appropriate time to stop training. After approximately 35 epochs in the beak dataset, the convergence trend of most models slowed. After about 100 epochs, the minimum value of the loss function was attained and the optimal fitting result was obtained.

This experiment used different CNN models for performance comparison on the testing set for the beaks, including VGG16, InceptionV3, ResNet18, ResNet32, ResNet50, and Resnet101.

**Figure 8:**
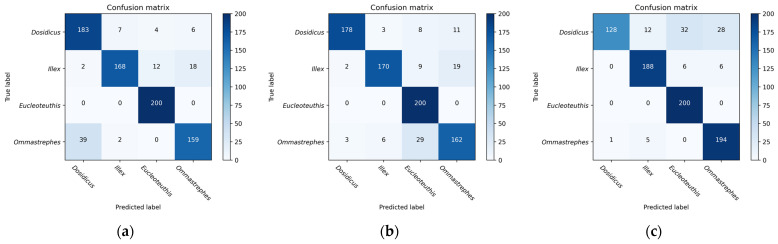

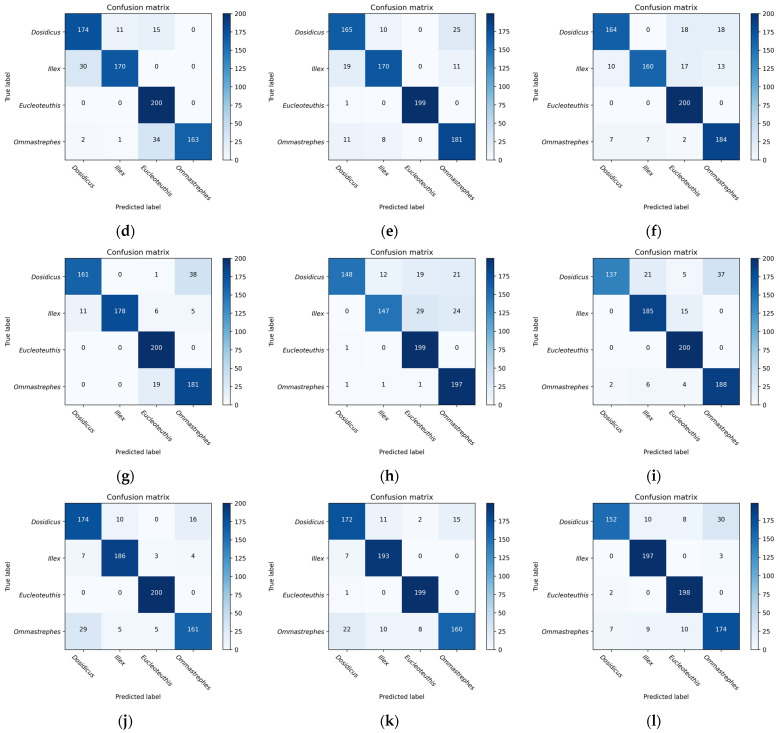
Confusion matrix for the six deep feature extraction models namely VGG16, InceptionV3, Resnet18, Resnet34, Resnet50, and Resnet101: (**a**–**f**) represents the confusion matrix for the upper beak; (**g**–**l**) represents the confusion matrix for the lower beak.

The recognition performance of the six CNN models on beaks was excellent, with accuracy ranging between 89.38% and 90.50% (Table 6). In contrast to the other four CNN models, InceptionV3 and Resnet18 had a recognition accuracy error of about 3.00% in the lower beak, and their accuracy was 86.40% and 87.60%, respectively. Resnet50 showed better classification abilities, with accuracy rates of 89.38% and 90.50% in the upper and lower beaks. The correct identification numbers of the upper beaks of *D. gigas*, *I. argentinus*, *E. luminosa*, and *O. bartramii* were 165, 170, 199, and 181, respectively. And the right number of species for the lower beaks was 172, 193, 199, and 160, respectively (Figure 8). In comparison to the confusion matrix of the other five models, Resnet50 had a more uniform and stable distribution of the number of correct recognitions in the four species of beaks, and the recognition rate was optimal overall. In particular, all of the CNN models successfully classified *E. luminosa*. The lower beaks performed better than the upper beaks following a comprehensive evaluation of the six CNN models.

### 3.3. Experimental Analysis of the Fusion of Shallow Features with Deep Features

The experiment examined ResNet50 as the backbone network for extracting deep features and fusing them with shallow features, and SVM was used for classification to test the performance of the fused features. The fully connected layer was the result of the multi-layer convolution of the obtained beak features. The feature information of this layer structure was very plentiful. There is a practical reason for extracting the feature vectors of the fully connected layer as parameter inputs to the SVM [22]. Therefore, the deep features of the fusion model originate from the fully connected layer of Resnet50, and the dimensions of the two fusion features are detailed (Table 3). 

**Figure 9:**
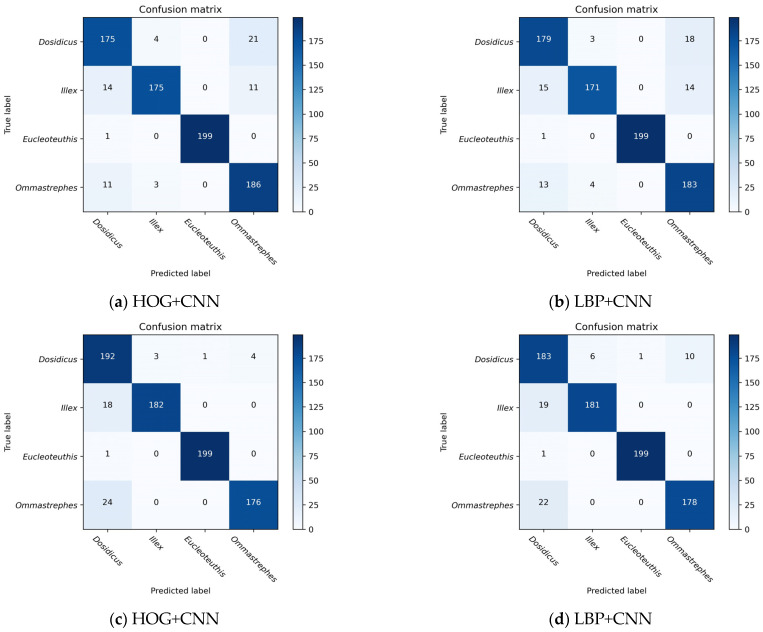
The confusion matrix of the fusion of deep features and shallow features: (**a**,**b**) represent the confusion matrix of the upper beak; (**c**,**d**) represent the confusion matrix of the lower beak.

Based on the results of the shallow experiments, the morphological features extracted by the HOG descriptor were used for feature fusion when C = 32. When R = 1 and P = 8, the texture features extracted by the LBP descriptor were used for feature fusion. The four species of beaks can be effectively classified using this method of feature fusion (Table 7 and Table 8). The results of the confusion matrix based on the CNN model and the feature fusion model (Figure 7 and Figure 9) indicate that the number of correct classifications of beaks can be significantly increased when feature fusion is applied. The feature fusion of HOG and CNN obtains the highest classification performance, with the average testing accuracy of 91.88% and 93.63% for the upper and lower beaks, respectively (Table 7 and Table 8). The correct identification numbers of the upper beaks of *D. gigas*, *I. argentinus*, *E. luminosa*, *O*. *bartramii* are 175, 175, 199, and 186, respectively. And the right number of species for the lower beaks is 192, 182, 199, and 176, respectively. Compared with the CNN model using deep features, the average testing accuracy of HOG+CNN for upper and lower beaks improved by 2.50% and 3.13%, respectively. And the average testing accuracy of LBP+CNN improved by 2.21% and 2.33%, respectively. In both feature fusion models, the accuracy of classification for *D. gigas*, *I. argentinus*, *E. luminosa*, and *O. bartramii* was improved in the upper beak. The classification accuracy of the lower beak of *D. gigas* and *O. bartramii* was improved. However, the classification accuracy of *I. argentinus* decreased.

## 4. Discussion

### 4.1. The Descriptors of the Two Local Shallow Features Are HOG and LBP

Morphological features and texture features are important shallow features in the study of image classification. If efficient morphological and textural features can be extracted, this is advantageous for beak classification. The HOG descriptor modifies the cell units to alter the range of local operations. After a comparison of classification experiments using three C values to extract morphological features, the lower beak achieved the highest classification accuracy of 60.50% when C = 32. However, the upper beak achieved the highest classification accuracy of 70.25% when C = 64. The morphological features obtained at C = 32 were used in the experiments for feature fusion. LBP was proposed to extract texture features. The texture features of the beak were extracted utilizing an enhanced circular LBP descriptor, with R and P values representing the number of the neighborhood radius and sample points, respectively. Three sets of different fusions of R and P values were employed, and the results demonstrate that the small neighborhood range was more appropriate for expressing the detailed features of the beak images. 

According to the results of the shallow feature experiments, morphological features were more effective than texture features in distinguishing the beaks of the four cephalopod species. The significant difference in the dimension of features retrieved by HOG and LBP is due to the HOG descriptor having an advantage in extracting high dimensional morphological features, which may convey and characterize variations in the details of the beak. The morphological specificity of the beaks [40] is superior in cephalopod biometrics, and the detailed variation within the two-dimensional morphology of the beak is extremely rich. Therefore, morphological features can be accurately extracted by analyzing image pairs from different perspectives [41]. There was some variation among the beak profile characterization factors of various squids, but they all contained several important characterization factors, such as upper hood length (UHL), upper crest length (UCL), and lower hood length (LHL), lower crest length (LCL), which indirectly provided a basis for the identification of cephalopod species using beak feature factors [10,42,43]. In order to meet the predatory needs and changes in the cephalopods during different growth periods, the pigmentation of the beaks also changes [43,44,45]. In addition, there were differences in pigmentation between male and female individuals [46]. Therefore, these factors also increase the difficulty of extracting discriminative texture features of similar beaks. The experimental results of feature fusion show that the HOG+CNN can improve classification accuracy. Therefore, we can infer that morphological features are more suitable for distinguishing beaks. 

### 4.2. CNN Model to Extract Global Deep Features

VGG16, InceptionV3, and Resnet series were used to extract the deep features of the beaks for performance comparison. Most models have a significant decrease in loss values at the start of training, indicating a suitable learning rate and gradient descent. After a certain stage of learning, the change in loss is not as obvious as at the beginning, and the loss curve tends to stabilize. Four evaluation metrics were used to assess the models, and all models were effective in extracting features and performed well in classification. VGG16 builds a deep network structure by stacking 16 convolutional layers, which is simple to understand and implement. However, VGG16 contains a huge number of parameters, which results in significant computational costs for training and inference. According to the loss function curve and evaluation indicators, VGG16 is easier to train on beaks but performs poorly in the upper beak classification of *D. gigas*. InceptionV3 improves the performance of image classification by introducing a structure of multi-scale feature extraction and parallel operation. InceptionV3 performs poorly on the beaks of *D. gigas* and *I. argentinus*. ResNet19 and ResNet34 are both equipped with skip connections and fewer full connection layers. Therefore, ResNet19 and Resnet34 have fewer parameters and faster convergence during training. Resnet18 performed the worst in the upper and lower beak classification of *D. gigas*. Resnet50 has a deeper network structure to acquire more complex and abstract feature representations, which achieves the highest classification accuracy in both the upper and lower beaks. Resnet101 is less accurate than Resnet50 since the beak dataset was too small to effectively train the Resnet101 model.

### 4.3. Advantages of Feature Fusion

The approach of fusing global deep features and local shallow features was employed in the classification of beaks for two key reasons. The first reason is that there are subtle interclass variations as well as large intraclass variations among species of beaks, which renders it challenging to classify specific regions based on subtle differences, and factors such as the morphology, size, pigmentation, age, and growth environment of the same species of cephalopod may all lead to differences. Therefore, the information contained in the fused features of different species can complement each other to produce a more robust feature representation, and the feature design and interpretation of shallow feature descriptors as well as the learning ability and generalization performance of deep learning can be used to improve accuracy in practical applications. Secondly, a strong advantage of deep learning is feature learning, i.e., automatic feature extraction from raw data, with features from higher levels of the hierarchy being formed by the composition of lower level features [36]. However, some species of beak samples are extremely challenging to acquire and belong to the category of tiny samples. As a result, the accurate identification of beaks using deep learning techniques is limited. Based on the global deep features, using local shallow features as an important reference for the classification task can effectively help in beak recognition. The results show that the fusion of deep features and shallow features can better represent the detailed features to distinguish the four beaks, compared to single deep features or shallow features. In particular, the HOG+Resnet50 model can more accurately show the distinctions between the beaks.

### 4.4. Using Multi-Class SVM Classifier for Beak Classification

The recognition results achieved by fused features for beaks of the same family but distinct genera using a multi-class SVM classifier were analyzed. Approximately 800 images of each kind of beak were used for training in feature extraction process. Due to the limited training samples, there may be overfitting and underfitting. SVM can perform nonlinear classification on small samples and enhance classifier performance by mapping data to high dimensional feature spaces using kernel functions. Second, SVM is insensitive to a small number of outliers or noise data and thus can handle interference effectively. During the experiment, the parameter C was adjusted to balance the fitting ability and generalization ability of the model. In summary, SVM has superior generalization ability, robustness, and controlled complexity, which can effectively solve classification problems in small sample datasets.

## 5. Conclusions

The study proposes an effective method for beak identification that fuses global deep features with local shallow features and uses multi-class SVM for automatic classification. In two shallow feature experiments, adjusting the parameters resulted in the greatest results, the HOG descriptor gave better results than the LBP descriptor in extracting the features. In CNN model experiments, Renet50 performed the best, achieving an upper beak accuracy of 89.38% and a lower beak accuracy of 90.50%. In the feature fusion experiments, both sets of fusion models showed good performance, and the feature fusion method of Resnet50+HOG achieved the highest recognition accuracy, with 91.88% and 93.63% for the upper and lower beaks, respectively. Resnet50+LBP achieved 91.50% and 92.63% for the upper and lower beak test datasets, respectively. Also, it was demonstrated that classification of the beak dataset can be effectively performed automatically using the multi-class SVM classifier. This study verifies the complementarity and differentiality of different types of features in the beak recognition task by using different performance analysis methods. The comparative analysis of fusion of different features shows that the fused features can be used to analyze the biodiversity of cephalopod beaks. Extracting HOG features, LBP features, deep features, and combining two types of features is conducive to the analysis of beaks, enriching the toolbox for studying cephalopod biology, and advancing the field of cephalopod biology. The combination of feature fusion with SVM-based recognition methods demonstrates robust performance. This not only promotes the automation of beak recognition but also fosters interdisciplinary collaboration and research by bridging deep learning, machine learning, and biological studies. Therefore, this approach drives the automation of beak recognition and provides an efficient and innovative research method applicable not only to cephalopods but also to various other biological domains. Since this study used a lower resolution image dataset and a more complex image background to classify beaks, the accuracy achieved by this research method can be applied. High-quality images will help to apply the research method more accurately to solve classification problems in the future. There are still many things we can achieve in cephalopod classification. Future research will continue to focus on the use of more beneficial shallow and deep features to obtain feature information of the beaks, and how to improve the usefulness of automatic classification tools to achieve the ultimate goal of real-time image processing.

## Figures and Tables

**Table 1:** Sampling information for four cephalopod samples.

Species	Sampling Location	Sampling Date
*Dosidicus gigas*	89° W~118° W, 0°~6° S	March 2020
*Illex argentinus*	57° W~60° W, 41° S~47° S	March 2021
*Eucleoteuthis luminosa*	158° E~162° E, 36° N~38° N	June 2021
*Ommastrephes bartramii*	157° E~164° E, 36° N~45° N	July 2022

**Table 2:** Parameters set in feature extraction and classification.

Model	Parameter Name	Parameter Value
SVM	K	‘rbf’
	C	10
	decision function morphological	OVR
CNN	learning rate	“1 × 10^−3^/1 × 10^−4^”
	epoch	100
	batch size	16

**Table 3:** The size of the input image, parameter settings, and vector dimensions of the features.

Models	Input	Parameter	Number of Feature Vectors
LBP	512 × 512	R = 1, P = 8	59
		R = 2, P = 8	59
		R = 2, R = 16	59
HOG	256 × 256	C = 16	8100
		C = 32	1764
		C = 64	324
Resnet50	224 × 224	FC	2048
LBP+Resnet50		FC, R = 1, P = 8	2107
HOG+Resnet50		FC, C = 32	3812

**Table 4:** Adjusting the R and P values in the LBP descriptor to obtain testing accuracy.

Testing Accuracy of LBP			
	R = 1, P = 8	R = 2, P = 8	R = 2, P = 16
Upper beak	53.63%	33.75%	30.88%
Lower beak	41.88%	40.88%	33.88%

**Table 5:** Adjusting the C value in the HOG descriptor to obtain testing accuracy.

Testing Accuracy of HOG			
	C = 16	C = 32	C = 64
Upper beak	65.25%	69.38%	70.25%
Lower beak	59.50%	60.50%	58.00%

**Table 6:** Accuracy for CNN models recognizing upper and lower beaks.

	Testing Accuracy of CNN Models	
Model	Upper Beak	Lower Beak
VGG16	88.75%	90.00%
InceptionV3	88.75%	86.40%
Resnet18	88.75%	87.60%
Resnet34	88.38%	90.13%
**Resnet50**	**89.38%**	**90.50%**
Resnet101	88.50%	90.13%

**Table 7:** Comparison of feature fusion experiments of the upper beak.

Models	Species	Accuracy	Precision	Recall	*F*1-*Score*
LBP+Resnet50	ALL	**91.50%**	**91.81%**	**91.50%**	**91.65%**
	*D. gigas*	89.50%	86.06%	89.50%	87.75%
	*I. argentinus*	85.50%	96.07%	85.50%	90.48%
	*E. luminosa*	99.50%	100.00%	99.50%	99.75%
	*O. bartramii*	91.50%	85.12%	91.50%	88.19%
HOG+Resnet50	ALL	**91.88%**	**92.13%**	**91.88%**	**92.00%**
	*D. gigas*	87.50%	87.06%	87.50%	87.28%
	*I. argentinus*	87.50%	96.15%	87.50%	91.62%
	*E. luminosa*	99.50%	100.00%	99.50%	99.75%
	*O. bartramii*	93.00%	85.32%	93.00%	88.99%

**Table 8:** Comparison of feature fusion experiments of the lower beak.

Models	Species	Accuracy	Precision	Recall	*F*1-*Score*
LBP+Resnet50	ALL	**92.63%**	**9** **3.08%**	**92.63%**	**92.85%**
	*D. gigas*	91.50%	81.33%	91.50%	86.12%
	*I. argentinus*	90.50%	96.79%	90.50%	93.54%
	*E. luminosa*	99.50%	99.50%	99.50%	99.50%
	*O. bartramii*	89.0%	94.68%	89.0%	91.75%
HOG+Resnet50	ALL	**93.63%**	**94.34%**	**93.63%**	**93.98%**
	*D. gigas*	96.00%	81.70%	96.00%	88.27%
	*I. argentinus*	91.00%	98.38%	91.00%	94.55%
	*E. luminosa*	99.50%	99.50%	99.50%	99.50%
	*O. bartramii*	88.00%	97.78%	88.00%	92.63%

## Data Availability

The data that support the findings of this study are available from the corresponding author upon reasonable request.
